# Atherogenic Index of Plasma for the Prediction of Delirium in ICU Patients: A Retrospective Cohort Study Based on the MIMIC‐IV Database

**DOI:** 10.1002/brb3.71613

**Published:** 2026-07-15

**Authors:** Shuai Miao, Xuyang Chen, Tianjun Wu, Yiling Qian, Jingjing Xu, Xin Zhang

**Affiliations:** ^1^ Department of Anesthesiology, The Affiliated Wuxi People's Hospital of Nanjing Medical University, Wuxi People's Hospital, Wuxi Medical Center Nanjing Medical University Wuxi Jiangsu Province China

**Keywords:** atherogenic index of plasma, delirium, ICU, MIMIC

## Abstract

**Background:**

This cohort study aimed to assess the association between atherogenic index of plasma (AIP) and the risk of delirium in intensive care unit (ICU) patients.

**Methods:**

Using the Medical Information Mart for Intensive Care IV (MIMIC‐IV) database, we analyzed 2,104 adult patients with ICU stays of ≥48 h. The relationship between AIP and delirium risk was investigated using logistic regression and restricted cubic splines (RCS). Subgroup analyses and interaction tests were conducted to assess effect modification. The primary endpoint was the incidence of delirium, with results expressed as odds ratio (OR) and 95% confidence intervals (CI).

**Results:**

After adjusting for confounding variables, elevated AIP was significantly associated with delirium risk (OR 1.48, 95% CI 1.15–1.91, *p* = 0.003). RCS analysis revealed a J‐shaped nonlinear relationship (*P* for nonlinearity = 0.01) between AIP and delirium with an inflection point at AIP = 0.33. Subgroup analyses confirmed consistent associations across demographic and clinical strata (all *P* for interaction > 0.05).

**Conclusion:**

This study suggests that AIP may serve as an independent biomarker for delirium risk in ICU patients, indicating its potential utility for early identification of high‐risk patients. The nonlinear association highlights the need for personalized risk assessment based on AIP values.

## Graphical abstract

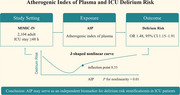

## 1 Introduction

Delirium, a common acute brain dysfunction in intensive care unit (ICU) patients, manifests as inattention, altered consciousness, and cognitive fluctuations (Cavallazzi et al. 2012). Studies indicate that ICU delirium affects 30% to 80% of patients and is strongly correlated with adverse outcomes, such as extended hospital stays (Kerber et al. 2020) and elevated mortality rates (Mokhtari et al. 2020). To effectively predict the incidence of delirium, researchers have proposed various potential biomarkers (Khan et al. 2013; Zhu et al. 2017; Kim et al. 2023) and clinical indicators (Cheng et al. 2023; Wang et al. 2023; Frost et al. 2024), such as C‐reactive protein (CRP) (Shyam et al. 2023), serum galectin‐3 levels (Zhu et al. 2017), and frailty. The integration of these biomarkers and assessment tools may enhance early detection and intervention strategies, ultimately improving patient prognosis.

The atherogenic index of plasma (AIP) has emerged as a robust biomarker for predicting cardiovascular outcomes, such as adverse cardiovascular events, ischemic stroke, and mortality across diverse populations (Ulloque‐Badaracco et al. 2022; Wu et al. 2024; Zheng et al. 2022; Rashidian et al. 2025; Zheng et al. 2023). In addition, growing evidence suggests that AIP may also be associated with kidney dysfunction, diabetic complications, and even cognitive impairment (Chen et al. 2025; Cui et al. 2025; Wei et al. 2026; Deng et al. 2025). Delirium shares several key pathological pathways with atherosclerotic progression, including systemic inflammation, oxidative stress, and microcirculatory disturbances. Given this background, it seems reasonable to hypothesize that AIP might also be associated with delirium risk. However, few studies have directly investigated this potential association. Thus, exploring the relationship between AIP and delirium may offer new insights into early risk assessment and targeted preventive strategies.

This study investigated the association between AIP and delirium risk in ICU patients using the Medical Information Mart for Intensive Care IV (MIMIC‐IV) database. To validate the consistency of this relationship, we performed comprehensive subgroup analyses stratified by key demographic and clinical variables, including age, sex, body mass index (BMI), diabetes, and hypertension.

## 2 Materials and Methods

### 2.1 Data Sources and Study Design

This retrospective cohort study utilized data extracted from the MIMIC‐IV database (Johnson et al. 2023), a comprehensive database containing information from more than 190,000 patients admitted to the ICU at Beth Israel Deaconess Medical Center between 2008 and 2019. One of the authors, Shuai Miao, completed the necessary training and certification process (certification number 13667135) to gain authorized access to the database. As the MIMIC‐IV database is a publicly accessible database, the Institutional Review Board of Beth Israel Deaconess Medical Center waived the requirement for informed consent. The study adhered to the Strengthening the Reporting of Observational Studies in Epidemiology (STROBE) guidelines to ensure rigorous reporting standards (von Elm et al. 2007).

### 2.2 Study Population

ICU patients were included in the study if they met the following criteria: (1) aged ≥18 years; (2) ICU stays of ≥48 h; (3) had triglyceride (TG) and high‐density lipoprotein cholesterol (HDL‐C) data for calculation of AIP; and (4) a primary diagnosis of delirium, as defined using International Classification of Diseases (ICD) codes, including both ICD‐9 and ICD‐10. Patients were excluded if (1) their TG or HDL‐C data were unavailable during their ICU stay or (2) they had pre‐existing dementia or neurocognitive disorders. In the case of patients with multiple admissions, only the first ICU admission record was considered to ensure accuracy.

### 2.3 Data Extraction

The author (SM) conducted data extraction from the MIMIC‐IV database using the PostgreSQL tool (version 14, PostgreSQL Global Development Group, Berkeley, California, USA). Variables extracted from the first day of ICU admission included (1) demographic information: age, sex, BMI, Glasgow Coma Scale score, Sequential Organ Failure Assessment score, length of ICU stay, and length of hospital stay; (2) comorbidities: hypertension, diabetes, myocardial infarction, chronic pulmonary disease, and atrial fibrillation; and (3) laboratory parameters: hemoglobin, hematocrit, red blood cell count, white blood cell count, platelet count, blood urea nitrogen, creatinine, blood glucose, sodium level, calcium level, chloride level, activated partial thromboplastin time, prothrombin time, alanine aminotransferase level, aspartate aminotransferase level, total bilirubin level, international normalized ratio, alkaline phosphatase level, TG level, and HDL‐C level. As this study was a retrospective analysis utilizing existing data from the MIMIC‐IV database, no prior statistical power calculation was performed, and the sample size was determined by the available data in the database. Variables with more than 20% missing data were excluded from the analysis to ensure data reliability. No missing data were present for categorical variables. Missing continuous variables were imputed using multiple imputation implemented in R software.

The primary exposure in this study was AIP, which was calculated using the formula: lg[TG(mmol/L)/HDL‐C(mmol/L)] (Onat et al. 2010). Patients were stratified into quartiles based on AIP, and outcomes were analyzed across these quartiles. The primary outcome was delirium during the ICU stay, diagnosed using the CAM‐ICU (Ely et al. 2001). Secondary outcomes included the length of ICU stay and length of hospital stay.

### 2.4 Statistical Analysis

Statistical analyses were conducted using R software. Continuous variables were presented as median (interquartile range) and analyzed using the Mann–Whitney *U* test. Categorical variables were presented as frequencies (percentages) and were analyzed using the chi‐square test or Fisher's exact test where applicable. Logistic regression models were fitted to evaluate the association between AIP and delirium. Covariates with *p* < 0.05 in univariate analysis were entered into the multivariable model. AIP was examined both as a continuous variable and as quartiles, and results were expressed as odds ratios (OR) with 95% confidence intervals (CI). Restricted cubic splines (RCS) with four knots (fifth, 35th, 65th, and 95th percentiles) were used to assess nonlinear relationships. Subgroup analyses were performed, stratified by age (<65 vs. ≥65 years), sex, BMI (<28 vs. ≥28 kg/m^2^), hypertension, and diabetes, with interaction terms tested using the likelihood ratio tests. A two‐sided *p* < 0.05 was considered statistically significant.

## 3 Results

### 3.1 Clinical Characteristics of Patients

**FIGURE 1:**
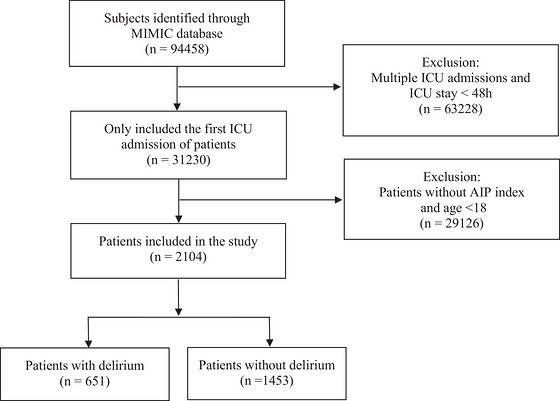
Flowchart of study participants. Abbreviations: AIP, Atherogenic index of plasma; MIMIC, Medical Information Mart for Intensive Care.

A total of 2104 patients from the MIMIC‐IV database were included in the analysis (Figure 1). The mean age was 66.76 years, and 60.98% were male. The median lengths of ICU and hospital stays were 5.66 and 12.87 days, respectively. Of the included patients, 651 (30.94%) developed delirium during their ICU stay. Significant differences between delirium and non‐delirium groups were observed for age, glucose levels, hematocrit, hemoglobin, white blood cell count, red blood cell count, creatinine, sodium, blood urea nitrogen, international normalized ratio, prothrombin time, alkaline phosphatase, and the prevalence of diabetes (all *p* < 0.05; Table 1). Furthermore, patients were stratified into AIP quartiles (Q1‐Q4) (Table 2). The incidence of delirium was 29.66%, 24.14%, 29.47%, and 40.49% in Q1 through Q4, respectively, with longer ICU and hospital stays in higher quartiles.

**TABLE 1:** Characteristics and outcomes of the delirium and non‐delirium groups.

Variables	Total (*n* = 2104)	Non‐delirium (*n* = 1453)	Delirium (*n* = 651)	*p*‐value
Age (years)	66.73 (56.48, 77.18)	66.48 (55.56, 76.82)	67.37 (58.30, 78.29)	0.037
Male, *n* (%)	1283 (60.98)	880 (60.56)	403 (61.90)	0.560
BMI	28.26 (24.48, 32.87)	27.99 (24.48, 32.76)	28.97 (24.49, 33.08)	0.172
GCS	15.00 (15.00, 15.00)	15.00 (15.00, 15.00)	15.00 (15.00, 15.00)	0.667
Glucose_max (mg/dL)	169.00 (133.00, 233.00)	165.00 (131.00, 224.00)	177.00 (138.25, 249.75)	< 0.001
Hematocrit _max (%)	38.10 (33.30, 42.60)	38.50 (33.70, 42.80)	36.80 (32.00, 42.08)	< 0.001
Hemoglobin_max (g/dL)	12.50 (10.88, 14.10)	12.70 (11.00, 14.20)	12.10 (10.30, 13.78)	< 0.001
Platelets_max (K/uL)	235.00 (176.00, 309.00)	238.00 (179.50, 308.00)	227.00 (169.50, 312.00)	0.516
White blood cell_max(K/Ul)	14.20 (10.50, 18.80)	13.90 (10.30, 18.20)	15.10 (11.10, 19.70)	< 0.001
Red blood cell_max (m/Ul)	4.14 (3.64, 4.67)	4.20 (3.71, 4.70)	4.00 (3.56, 4.56)	< 0.001
Creatinine_max (mg/dL)	1.20 (0.90, 1.90)	1.10 (0.90, 1.70)	1.40 (1.00, 2.50)	< 0.001
ALT_max (IU/L)	34.00 (19.00, 87.00)	32.00 (18.00, 80.25)	38.00 (19.00, 100.00)	0.036
AST_max (IU/L)	51.00 (27.00, 161.75)	48.00 (26.00, 151.50)	57.00 (28.00, 167.25)	0.024
Total bilirubin_max (mg/dL)	0.70 (0.50, 1.30)	0.70 (0.50, 1.20)	0.80 (0.50, 1.58)	0.173
Sodium _max (mEq/L)	141.00 (138.00, 144.00)	141.00 (138.00, 144.00)	142.00 (139.00, 145.75)	< 0.001
Calcium _max (mg/dL)	8.90 (8.40, 9.30)	8.90 (8.50, 9.30)	8.90 (8.40, 9.30)	0.757
Bun_max (mg/dl)	24.00 (16.00, 41.00)	22.00 (15.00, 36.00)	30.00 (19.00, 52.00)	< 0.001
Chloride_max (mEq/L)	106.00 (103.00, 110.00)	106.00 (103.00, 110.00)	107.00 (103.00, 111.00)	0.067
INR_max	1.30 (1.20, 1.80)	1.30 (1.10, 1.70)	1.40 (1.20, 1.92)	< 0.001
PT_max (seconds)	14.60 (12.70, 19.20)	14.30 (12.60, 18.30)	15.50 (13.20, 21.10)	< 0.001
APTT Max (seconds)	36.20 (28.90, 71.05)	35.80 (28.65, 69.90)	37.15 (29.48, 72.15)	0.051
ALP _max (U/L)	87.00 (66.00, 123.00)	85.00 (66.00, 116.00)	91.00 (68.00, 137.00)	< 0.001
Triglyceride_max (mg/dl)	1.34 (0.95, 2.10)	1.32 (0.93, 1.99)	1.43 (0.98, 2.36)	0.001
HDL_max (mg/dl)	0.66 (0.49, 0.87)	0.68 (0.52, 0.87)	0.61 (0.44, 0.87)	< 0.001
AIP	0.33 (0.09, 0.58)	0.30 (0.08, 0.54)	0.40 (0.10, 0.70)	< 0.001
Diabetes, *n* (%)	537 (25.52)	326 (22.44)	211 (32.41)	< 0.001
Hypertension, *n* (%)	692 (32.89)	468 (32.21)	224 (34.41)	0.321
MI, *n* (%)	348 (16.54)	219 (15.07)	129 (19.82)	0.007
AF, n (%)	303 (14.40)	210 (14.45)	93 (14.29)	0.920
COPD, n (%)	193 (9.17)	122 (8.40)	71 (10.91)	0.065
Sepsis, n (%)	1985 (94.34)	1345 (92.57)	640 (98.31)	< 0.001
SOFA	6.00 (4.00, 9.00)	6.00 (3.00, 9.00)	7.00 (5.00, 11.00)	< 0.001
ICU Los (day)	5.66 (3.36, 10.49)	5.14 (3.15, 9.73)	6.84 (3.92, 12.71)	< 0.001
Hospital Los (day)	12.87 (7.57, 23.18)	10.85 (6.55, 20.63)	17.12 (10.72, 29.81)	< 0.001

*Note*: Values are expressed as median (interquartile range) or number of patients (%). Abbreviations: AF, Atrial fibrillation; AIP, atherogenic index of plasma; ALP, alkaline Phosphatase; ALT, Alanine Aminotransferase; APTT, activated partial thromboplastin time; AST, Aspartate Aminotransferase; BMI, body mass index; BUN, blood urea nitrogen; COPD, chronic obstructive pulmonary disease; GCS, Glasgow coma scale; HDL, high‐density lipoprotein; Hospital Los, hospital length of stay; ICU Los, intensive care unit length of stay; INR, International Normalized Ratio; MI, myocardial infarction; PT, prothrombin time; SOFA, sequential organ failure assessment.

**TABLE 2:** Characteristics and outcomes of participants categorized by AIP.

Variables	Total (*n* = 2104)	Q1 (*n* = 526)	Q2 (*n* = 526)	Q3 (*n* = 526)	Q4 (*n* = 526)	*p*‐value
Age (years)	66.73 (56.48, 77.18)	70.63 (60.06, 81.58)	69.88 (61.05, 80.18)	65.25 (55.29, 75.35)	61.33 (50.70, 70.27)	< 0.001
Male, *n* (%)	1283 (60.98)	285 (54.18)	323 (61.41)	314 (59.70)	361 (68.63)	< 0.001
BMI	28.26 (24.48, 32.87)	26.91 (23.16, 30.50)	27.47 (24.19, 31.79)	28.78 (24.93, 33.70)	30.29 (25.89, 34.62)	< 0.001
GCS	15.00 (15.00, 15.00)	15.00 (15.00, 15.00)	15.00 (15.00, 15.00)	15.00 (15.00, 15.00)	15.00 (15.00, 15.00)	< 0.001
Glucose_max (mg/dL)	169.00 (133.00, 233.00)	156.00 (128.00, 205.00)	163.00 (129.75, 223.00)	174.50 (136.00, 250.50)	183.00 (140.75, 261.00)	< 0.001
Hematocrit _max (%)	38.10 (33.30, 42.60)	38.70 (34.40, 42.60)	38.50 (34.30, 43.30)	38.15 (33.30, 42.68)	36.70 (31.80, 42.00)	<.001
Hemoglobin_max (g/dL)	12.50 (10.88, 14.10)	12.70 (11.30, 14.10)	12.70 (11.00, 14.10)	12.55 (10.70, 14.00)	12.10 (10.30, 13.90)	0.002
Platelets_max (K/uL)	235.00 (176.00, 309.00)	233.00 (182.00, 294.00)	231.00 (174.00, 297.00)	241.50 (181.25, 325.00)	237.00 (168.00, 326.00)	0.189
White blood cell_max(K/Ul)	14.20 (10.50, 18.80)	12.90 (9.90, 16.80)	13.90 (10.00, 17.60)	14.70 (10.90, 19.17)	16.40 (11.90, 21.70)	< 0.001
Red blood cell_max (m/Ul)	4.14 (3.64, 4.67)	4.13 (3.70, 4.62)	4.22 (3.71, 4.72)	4.19 (3.60, 4.77)	4.06 (3.59, 4.56)	0.005
Creatinine_max (mg/dL)	1.20 (0.90, 1.90)	1.10 (0.80, 1.50)	1.20 (0.90, 1.67)	1.20 (0.90, 1.90)	1.50 (1.00, 2.80)	< 0.001
ALT_max (IU/L)	34.00 (19.00, 87.00)	25.00 (17.00, 49.00)	31.00 (18.00, 73.00)	35.50 (19.00, 96.25)	52.50 (24.00, 156.25)	< 0.001
AST_max (IU/L)	51.00 (27.00, 161.75)	39.00 (24.00, 93.00)	43.00 (25.00, 127.00)	51.50 (27.00, 188.00)	86.00 (37.00, 255.00)	< 0.001
Total bilirubin_max (mg/dL)	0.70 (0.50, 1.30)	0.70 (0.50, 1.00)	0.70 (0.50, 1.20)	0.70 (0.50, 1.30)	0.90 (0.50, 2.20)	< 0.001
Sodium _max (mEq/L)	141.00 (138.00, 144.00)	141.00 (138.00, 144.00)	141.00 (138.00, 144.00)	141.00 (139.00, 144.00)	141.00 (138.00, 145.00)	0.134
Calcium _max (mg/dL)	8.90 (8.40, 9.30)	9.00 (8.60, 9.30)	8.90 (8.50, 9.40)	8.80 (8.50, 9.30)	8.70 (8.10, 9.10)	< 0.001
Bun_max (mg/dl)	24.00 (16.00, 41.00)	20.00 (15.00, 33.00)	24.00 (16.00, 36.75)	25.00 (17.00, 42.00)	30.00 (19.00, 51.00)	< 0.001
Chloride_max (mEq/L)	106.00 (103.00, 110.00)	106.00 (103.00, 109.00)	106.00 (103.00, 110.00)	107.00 (103.00, 110.00)	107.00 (103.00, 111.00)	0.009
INR_max	1.30 (1.20, 1.80)	1.20 (1.10, 1.50)	1.30 (1.20, 1.70)	1.40 (1.20, 1.80)	1.40 (1.20, 2.00)	< 0.001
PT_max (seconds)	14.60 (12.70, 19.20)	13.40 (12.10, 16.90)	14.50 (12.80, 18.60)	14.90 (13.00, 19.50)	15.80 (13.50, 21.48)	< 0.001
APTT Max (seconds)	36.20 (28.90, 71.05)	34.10 (28.50, 78.00)	35.70 (28.70, 71.00)	36.80 (29.00, 65.80)	38.20 (29.80, 67.15)	0.461
ALP _max (U/L)	87.00 (66.00, 123.00)	81.00 (63.50, 105.00)	84.00 (66.00, 116.00)	89.00 (67.50, 123.00)	99.00 (69.00, 151.00)	< 0.001
Triglyceride_max (mg/dl)	1.34 (0.95, 2.10)	0.79 (0.64, 0.96)	1.16 (0.97, 1.42)	1.69 (1.34, 2.07)	2.81 (2.10, 4.54)	< 0.001
HDL_max (mg/dl)	0.66 (0.49, 0.87)	1.00 (0.84, 1.18)	0.73 (0.63, 0.84)	0.59 (0.49, 0.72)	0.40 (0.26, 0.54)	< 0.001
AIP	0.33 (0.09, 0.58)	−0.09 (−0.19, 0.00)	0.21 (0.15, 0.26)	0.45 (0.39, 0.52)	0.83 (0.69, 1.12)	< 0.001
SOFA	6.00 (4.00, 9.00)	5.00 (3.00,7.00)	6.00 (4.00, 8.00)	6.00 (4.00, 9.00)	9.00 (6.00, 12.00)	< 0.001
Diabetes, *n* (%)	537 (25.52)	96 (18.25)	128 (24.33)	158 (30.04)	155 (29.47)	< 0.001
Hypertension, *n* (%)	692 (32.89)	189 (35.93)	166 (31.56)	172 (32.70)	165 (31.37)	0.364
MI, *n* (%)	348 (16.54)	82 (15.59)	92 (17.49)	97 (18.44)	77 (14.64)	0.328
AF, *n* (%)	303 (14.40)	87 (16.54)	83 (15.78)	69 (13.12)	64 (12.17)	0.133
COPD, *n* (%)	193 (9.17)	46 (8.75)	50 (9.51)	50 (9.51)	47 (8.94)	0.962
ICU Los (day)	5.66 (3.36, 10.49)	4.97 (3.00, 7.85)	4.78 (2.98, 9.12)	5.56 (3.55, 10.08)	8.33 (4.31, 15.48)	< 0.001
Hospital Los (day)	12.87 (7.57, 23.18)	9.52 (6.11, 16.08)	10.93 (6.68, 18.94)	13.55 (7.81, 24.36)	20.15 (11.09, 34.30)	< 0.001
Sepsis, *n* (%)	1985 (94.34)	481 (91.44)	493 (93.73)	503 (95.63)	508 (96.58)	0.002
Delirium, *n* (%)	651 (30.94)	156 (29.66)	127 (24.14)	155 (29.47)	213 (40.49)	< 0.001

*Note*: Values are expressed as median (interquartile range) or number of patients (%). Abbreviations: AF, atrial fibrillation; ALP, alkaline phosphatase; ALT, alanine aminotransferase; AIP, atherogenic index of plasma; APTT, activated partial thromboplastin time; AST, aspartate aminotransferase; BMI, body mass index; COPD, chronic obstructive pulmonary disease; GCS, Glasgow Coma Scale; HDL, high‐density lipoprotein; Hospital LOS, hospital length of stay; ICU LOS, intensive care unit length of stay; INR, international normalized ratio; MI, myocardial infarction; PT, prothrombin time; SOFA, sequential organ failure assessment.

### 3.2 Associations Between AIP and Delirium

Table 3 shows the logistic regression analysis for delirium. As a continuous variable, AIP was significantly associated with delirium in both univariate (OR 1.59, 95% CI 1.28–1.97, *p* < 0.001) and multivariable analyses (adjusted OR 1.48, 95% CI 1.15–1.91, *p* = 0.003). When analyzed by quartiles (with Q1 as reference), the adjusted ORs were 0.63 (0.46–0.86) for Q2, 0.88 (0.65–1.19) for Q3, and 1.39 (1.02–1.88) for Q4.

**TABLE 3:** The association between AIP groups and delirium.

Exposure	Model 1		Model 2	
	OR (95%CI)	*p*‐value	OR (95%CI)	*p*‐value
AIP as continuous	1.59 (1.28 ∼ 1.97)	< 0.001	1.48 (1.15 ∼ 1.91)	0.003
Q1	Ref		Ref	
Q2	0.75 (0.57 ∼ 0.99)	0.044	0.63 (0.46 ∼ 0.86)	0.004
Q3	0.99 (0.76 ∼ 1.29)	0.946	0.88 (0.65 ∼ 1.19)	0.414
Q4	1.61 (1.25 ∼ 2.08)	< 0.001	1.39 (1.02 ∼ 1.88)	0.035

Model 1: Unadjusted.

Model 1: Adjusted for age; diabetes; myocardial infarction; glucose; hematocrit; hemoglobin;; white blood cell; red blood cell; creatinine; alanine aminotransferase; aspartate aminotransferase; sodium;blood urea nitrogen; international normalized ratio; prothrombin time; alkaline phosphatase.

Abbreviations: AIP, atherogenic index of plasma; CI, confidence interval; OR, odds ratio.

### 3.3 Restricted Cubic Spline Analysis

**FIGURE 2:**
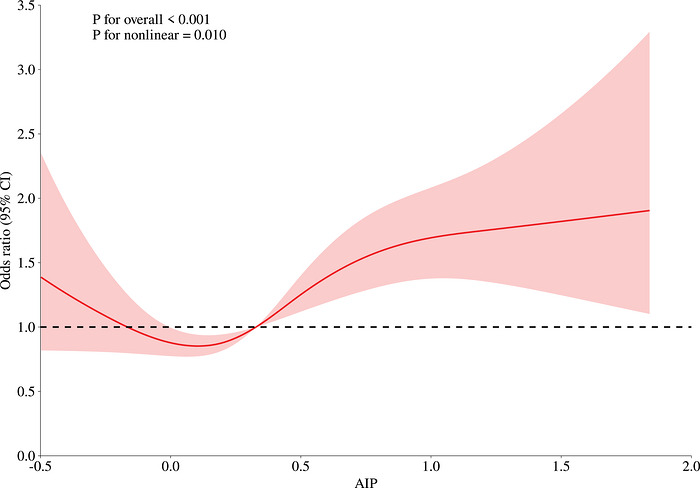
Restricted cubic spline for the relationship between the AIP and delirium in ICU patients.

RCS revealed a significant nonlinear positive association between AIP and delirium risk (*P* for nonlinearity = 0.01; Figure 2). The odds of delirium increased progressively with rising AIP with a notable inflection point at 0.33, beyond which risk escalated more sharply.

### 3.4 Subgroup Analysis

Subgroup analyses stratified by age, sex, BMI, diabetes, and hypertension showed no significant interactions (all *P* for interaction > 0.05; Table 4), confirming the robustness of the AIP‐delirium association across all subgroups.

**TABLE 4:** Subgroup analyses for the association of AIP with delirium.

Subgroup	N (%)	OR (95% CI)	*p*‐value	*P* for interaction
Overall	2104 (100.00)	1.59 (1.28 ∼ 1.97)	< 0.001	
Gender				0.967
Female	821 (39.02)	1.58 (1.09 ∼ 2.28)	0.015	
Male	1283 (60.98)	1.59 (1.22 ∼ 2.08)	< 0.001	
Age				0.312
<65	940 (44.68)	1.88 (1.38 ∼ 2.56)	< 0.001	
≥65	1164 (55.32)	1.50 (1.10 ∼ 2.04)	0.011	
BMI				0.599
<28	1018 (48.38)	1.43 (1.01 ∼ 2.04)	0.047	
≥28	1086 (51.62)	1.61 (1.22 ∼ 2.13)	< 0.001	
Diabetes				0.978
No	1567 (74.48)	1.54 (1.19 ∼ 1.98)	< 0.001	
Yes	537 (25.52)	1.53 (1.00 ∼ 2.33)	0.050	
Hypertension				0.514
No	1412 (67.11)	1.52 (1.16 ∼ 1.97)	0.002	
Yes	692 (32.89)	1.76 (1.22 ∼ 2.55)	0.003	

Abbreviations: AIP, atherogenic index of plasma; BMI, body mass index; CI, confidence interval; OR, odds ratio.

## 4 Discussion

This study systematically evaluated the association between AIP and delirium risk in ICU patients. Higher AIP levels independently predicted an increased risk of delirium, demonstrating a nonlinear relationship with an inflection point at 0.33. Specifically, AIP as a single biomarker yielded an adjusted odds ratio of 1.39 (95% CI: 1.19–1.63), indicating a modest but statistically independent effect. The association remained consistent across all subgroups, including age, sex, BMI, diabetes, and hypertension, as confirmed by comprehensive subgroup analyses. These findings position AIP as a potential biomarker for delirium risk stratification in critical care settings.

The nonlinear J‐shaped association observed in this study aligns with a growing body of evidence demonstrating threshold effects of metabolic biomarkers on clinical outcomes in critically ill populations. For instance, using the same MIMIC‐IV database, Lou et al. (2025) identified a critical threshold for the glucose‐potassium ratio (GPR) in septic patients, with a substantially increased mortality risk beyond GPR = 30. Collectively, these findings support the notion that metabolic biomarkers often exhibit threshold phenomena in critical illness: risk remains relatively stable below a certain level but escalates markedly once the threshold is exceeded. Such nonlinear relationships highlight the utility of risk stratification based on biomarker thresholds rather than linear models, which may underestimate risk at higher levels. Although speculative, given that causality cannot be determined, the J‐shaped inflection point at AIP = 0.33 might reflect a shift from compensatory to decompensated lipid dysregulation. Several plausible pathways have been proposed in the literature and warrant consideration as hypotheses for future investigation.

### 4.1 Cholesterol‐Serotonin Signaling Pathway

Emerging evidence suggests that cholesterol dysregulation may impair neurotransmitter systems, particularly serotonin signaling (Fantini et al. 2024; Aguiar and Giaquinto 2018; Massaccesi et al. 2020), which is implicated in various neuropsychiatric outcomes. The cholesterol‐serotonin hypothesis posits that reduced cholesterol levels, whether from dietary deficiencies or synthesis inhibition, may decrease central serotonin activity, potentially predisposing to mood disorders (Cheon 2023). Abnormal lipid metabolism significantly impacts brain physiology through multiple pathways (Toprak and Musselman 2021). Reduced HDL‐C levels and elevated LDL/HDL ratios have been consistently associated with increased delirium risk in ICU settings (Li et al. 2021; Sugimoto et al. 2015; Melamud et al. 2024). These lipid alterations may compromise cerebral perfusion through accelerated atherosclerosis while simultaneously affecting neuronal membrane integrity and neurotransmission efficiency. However, these pathways are speculative and derived primarily from non‐delirium studies.

### 4.2 Glucose Metabolism Dysregulation

AIP–delirium association may also be hypothetically mediated through glucose metabolism dysregulation. AIP strongly correlates with insulin resistance and impaired glucose homeostasis, potentially leading to hyperglycemia or glucose variability, both established risk factors for ICU delirium (Adamis and Eikelenboom 2022; Wang et al. 2023). Potential hyperglycemia‐induced delirium hypotheses include (1) increased cerebral oxidative stress (Redhwan et al. 2024) and neuroinflammation (Lee et al. 2024), (2) blood‐brain barrier disruption (Wei et al. 2023), and (3) impaired neurotransmitter release and synaptic plasticity (Pintana et al. 2017). Clinical studies demonstrate that glycemic variability significantly predicts delirium incidence, particularly in elderly ICU patients, while intensive glucose control reduces delirium risk (Choi et al. 2022). These findings suggest that AIP may influence delirium risk through pathways involving glucose regulation, but direct evidence linking these mechanisms to AIP–delirium association is lacking.

### 4.3 Gut‐Brain Axis and Intestinal Barrier Dysfunction

Elevated AIP, reflecting atherogenic dyslipidemia and insulin resistance, is associated with chronic low‐grade systemic inflammation that can compromise intestinal mucosal integrity. (Deng et al. 2022, Zhuang et al. 2021) AIP‐related metabolic dysregulation may impair PI3K/AKT signaling, critical for both intestinal epithelial repair and neuronal survival, thereby disrupting the gut‐brain axis via two parallel mechanisms: (1) increased gut permeability leading to bacterial translocation and systemic endotoxemia, thereby amplifying neuroinflammation; and (2) reduced neuronal protection, rendering the brain more vulnerable to inflammatory insults. This framework positions intestinal barrier integrity as a potential bridge between peripheral metabolic dysfunction and central vulnerability. However, direct testing using permeability markers and endotoxin levels is needed in future studies. These pathways likely converge on shared inflammatory and metabolic disturbances that predispose to delirium.

### 4.4 Comparison With Existing Markers

In the prediction and diagnosis of ICU delirium, AIP has been proposed as a potential biomarker with certain theoretical advantages over traditional markers, including inflammatory, oxidative stress, and neuroendocrine indicators. However, this study did not directly compare the predictive performance of AIP with these markers, and claims regarding its utility remain preliminary. First, unlike acute‐phase inflammatory markers such as CRP and interleukin‐6 (IL‐6), which reflect short‐term inflammatory responses (Imai et al. 2023; Zhang et al. 2024), AIP primarily reflects lipid metabolism abnormalities and may capture chronic metabolic vulnerability. While elevated CRP and IL‐6 have been associated with delirium in infectious or systemic inflammatory states, AIP may theoretically offer complementary information in patients with chronic metabolic abnormalities. Second, compared to oxidative stress markers such as malondialdehyde and superoxide dismutase, which require specialized laboratory techniques (Pang et al. 2022; Podgoreanu et al. 2020), AIP can be calculated from routine lipid profiles, potentially offering a simpler and more cost‐effective alternative. Third, unlike neuroendocrine markers such as cortisol (Zheng et al. 2022; Witlox et al. 2020) and norepinephrine (Phero et al. 2025; Jordano et al. 2024), which are influenced by acute stress responses, AIP reflects longer‐term metabolic status and may provide a different perspective on patient risk. Nevertheless, these potential advantages remain speculative in the absence of formal head‐to‐head comparisons. Whether AIP adds incremental predictive value beyond established clinical or laboratory predictors, or simply reflects overlapping information, has not been evaluated in this study. Future research using appropriate discrimination and reclassification metrics is needed to determine the clinical utility of AIP relative to existing markers.

### 4.5 Clinical Application Value

AIP may serve as a convenient, routinely available, low‐cost biomarker with incremental prognostic value for delirium risk stratification in ICU patients. This potential utility is reflected in four aspects: (1) capturing chronic metabolic vulnerability that complements acute inflammation; (2) providing a clear nonlinear inflection point (AIP = 0.33) for identifying elevated delirium risk; (3) remaining independent of traditional risk factors, with consistent findings across subgroups; and (4) offering simplicity and reproducibility, derived from standard lipid panels, calculable at the bedside within 24 h of ICU admission at no extra cost, which supports its feasibility as a low‐cost screening tool.

Nevertheless, several key steps are required before clinical implementation. Prospective multicenter validation is paramount to confirm the generalizability of the observed inflection point (AIP = 0.33) and the robustness of the J‐shaped association across diverse healthcare systems and ethnic populations. Standardized measurement protocols also need to be established, including uniform blood sampling timing (e.g., within 6 h of ICU admission) and documentation of confounders such as propofol infusion rates and nutritional support. Dynamic AIP monitoring should be explored, as serial measurements (daily or every 48 h) could help determine whether rising AIP precedes delirium onset or whether persistent elevation identifies patients at prolonged risk. Head‐to‐head comparisons with established delirium prediction tools are necessary to quantify incremental value. If the AIP‐delirium association is replicated, it could motivate interventional hypothesis generation, including proof‐of‐concept trials of lipid‐modifying interventions. Moreover, future studies should integrate emerging biomarkers to examine whether AIP adds predictive value beyond or in combination with inflammatory markers, neuronal injury markers, or gut barrier markers. Finally, from an implementation science perspective, if AIP is validated as a robust predictor, pragmatic studies should evaluate whether routine AIP reporting with an alert for values > 0.33 changes clinician behavior and improves patient outcomes. In summary, the path from discovery to clinical application requires rigorous prospective multicenter validation, standardization, dynamic assessment, and ultimately interventional and implementation trials.

### 4.6 Limitations

Despite its strengths, this study has several limitations. First, the analysis was restricted to a single‐center US ICU population, which limits the generalizability of findings to other settings such as non‐ICU patients or different healthcare systems and ethnic populations. Second, the retrospective design introduces the potential for unmeasured confounding, despite adjustments for available variables. Factors not recorded in MIMIC‐IV (e.g., nutritional status and frailty) may influence both AIP and delirium risk, and residual confounding cannot be entirely excluded. Third, the reliance on ICD codes for delirium diagnosis may introduce misclassification bias. The MIMIC‐IV database does not reliably distinguish delirium from metabolic or toxic encephalopathy, and CAM‐ICU has lower sensitivity for hypoactive delirium, particularly in older and medically complex patients. This under‐detection is likely non‐differential with respect to AIP levels, which would bias the observed association toward the null. However, if the under‐detection varies systematically with AIP levels (e.g., higher AIP patients being more prone to hypoactive delirium), the observed odds ratio could be either underestimated or overestimated. Without external data on AIP and delirium motor subtypes, the direction of bias remains uncertain. Similarly, if AIP is more strongly associated with metabolic encephalopathy than with pure delirium, outcome misclassification may further distort effect estimates.

## 5 Conclusions

This study suggests that AIP may be a potential biomarker for assessing delirium risk in ICU patients, though its clinical utility remains preliminary. While our findings add to the existing literature on delirium risk factors, further research is needed to validate these results in diverse populations, clarify the underlying mechanisms, and determine whether AIP offers incremental predictive value beyond current models. Expanding future investigations to include pre‐operative and post‐surgical settings will help further evaluate the role of AIP in delirium‐risk stratification.

## Author Contributions

**Xuyang Chen**: conceptualization, methodology, data curation, Writing – original draft, Writing – review and editing, resources. **Xin Zhang**: conceptualization, methodology, software, data curation, writing – original draft, writing – review and editing, funding acquisition, resources, supervision. **Tianjun Wu**: software, data curation. Yiling Qian: software, data curation. **Shuai Miao**: conceptualization, methodology, software, data curation, investigation, validation, formal analysis, supervision, visualization, project administration, writing – original draft, writing – review and editing. **Jingjing Xu**: software, data curation, writing – original draft, writing – review and editing, conceptualization, methodology.

## Funding

This work was supported by the National Natural Science Foundation of China (82271251 X.Z).

## Ethics Statement

This study was conducted using a publicly available, de‐identified database. Therefore, ethical approval and informed consent were waived.

## Conflicts of Interest

The authors declare no conflicts of interest.

## Data Availability

The data that support the findings of this study are available from a publicly accessible database (MIMIC‐IV, https://mimic.mit.edu). Access to the database was granted after completion of the required training course, and approval was obtained from the Institutional Review Boards of the Massachusetts Institute of Technology (MIT) and Beth Israel Deaconess Medical Center.
